# Supplementary material for: Comparative multiomics analysis of cell physiological state after culture in a basket bioreactor
Source: Sci Rep. 2022 Nov 23;12:20161. doi: 10.1038/s41598-022-24687-4 (PMC9686226; doi:10.1038/s41598-022-24687-4)
Supplement: Supplementary file 1 — Supplementary Information 1. [file 41598_2022_24687_MOESM1_ESM.zip › raw data/Proteomic raw data/6.KEGGenrichment/EG.vs.CG/ALL/pathway/src/05205.html]

map05205


- K04707

- Up regulated proteins

A0A0D9R1V2(Description:E3 ubiquitin-protein ligase CBL;Gene:CBLB;log2FC:1.1276)

- K03258

- Up regulated proteins

A0A0D9QZT6(Description:RRM domain-containing protein;Gene:EIF4B;log2FC:2.5466)

- K02235, K02329, K02354, K02375, K02376, K02432, K02842

- Up regulated proteins


A0A0D9SCP4(Description:Uncharacterized protein;Gene:FZD1;log2FC:1.4940)

- K02235, K02329, K02354, K02375, K02376, K02432, K02842

- Up regulated proteins


A0A0D9SCP4(Description:Uncharacterized protein;Gene:FZD1;log2FC:1.4940)

- K05717

- Down regulated proteins

A0A0D9R9X4(Description:Fibronectin;Gene:FN1;log2FC:-1.1754)

- K05717

- Down regulated proteins

A0A0D9R9X4(Description:Fibronectin;Gene:FN1;log2FC:-1.1754)

- K05717

- Down regulated proteins

A0A0D9R9X4(Description:Fibronectin;Gene:FN1;log2FC:-1.1754)

- K06255

- Down regulated proteins

A0A0D9S8B1(Description:Uncharacterized protein;Gene:HSPG2;log2FC:-2.5557)

- K06255

- Down regulated proteins

A0A0D9S8B1(Description:Uncharacterized protein;Gene:HSPG2;log2FC:-2.5557)

Close
